# Low Molecular Weight Barley β-Glucan Affects Glucose and Lipid Metabolism by Prebiotic Effects

**DOI:** 10.3390/nu13010130

**Published:** 2020-12-31

**Authors:** Seiichiro Aoe, Kento Mio, Chiemi Yamanaka, Takao Kuge

**Affiliations:** 1 Studies in Human Life Sciences, Graduate School of Studies in Human Culture, Otsuma Women’s University, Chiyoda-ku, Tokyo 102-8357, Japan; mio.kento@hakubaku.co.jp; 2 The Institute of Human Culture Studies, Otsuma Women’s University Chiyoda-ku, Tokyo 102-8357, Japan; chiemiy@gmail.com; 3 ADEKA Corporation, Tokyo 116-8553, Japan; kuge@adeka.co.jp

**Keywords:** barley, β-glucan, low molecular weight, fermentation, prebiotics

## Abstract

We investigated the effect of low molecular weight barley β-glucan (LMW-BG) on cecal fermentation, glucose, and lipid metabolism through comparisons to high molecular weight β-glucan (HMW-BG). C57BL/6J male mice were fed a moderate-fat diet for 61 days. LMW-BG or HMW-BG was added to the diet corresponding to 4% β-glucan. We measured the apparent absorption of fat, serum biomarkers, the expression levels of genes involved in glucose and lipid metabolism in the liver and ileum, and bacterial counts of the major microbiota groups using real time PCR. The concentration of short-chain fatty acids (SCFAs) in the cecum was analyzed by GC/MS. Significant reductions in serum leptin, total- and LDL-cholesterol concentrations, and mRNA expression levels of sterol regulatory element-binding protein-1c (SREBP-1c) were observed in both BG groups. HMW-BG specific effects were observed in inhibiting fat absorption and reducing abdominal deposit fat, whereas LMW-BG specific effects were observed in increasing bacterial counts of *Bifidobacterium* and *Bacteroides* and cecal total SCFAs, acetate, and propionate. mRNA expression of neurogenin 3 was increased in the LMW-BG group. We report that LMW-BG affects glucose and lipid metabolism via a prebiotic effect, whereas the high viscosity of HMW-BG in the digestive tract is responsible for its specific effects.

## 1. Introduction

Barley and oats are rich in β-glucan, which has several positive effects on inhibiting a postprandial glucose rise [1,2,3] and improvement in serum cholesterol concentrations [4,5,6,7,8]. Barley and oat β-glucans have β-1,3 and β-1,4 glycosidic polysaccharides with high molecular weight [9,10]. It is generally recognized that β-glucans in barley and oats with higher molecular weights are essential for a reduction in postprandial blood glucose rise and incremental area under the blood concentration curve (IAUC) [11,12,13]. It was also reported that an increase in excretion of neutral and acidic sterols reduced serum LDL cholesterol concentrations by promoting catabolism and reduced cholesterol absorption [14]. This mechanism is positively related to the high viscosity of β-glucan in the small intestine and consequent increase in the excretion of neutral and acidic sterols [15]. However, a previous study reported that the serum lipid profile in mice fed a high-fat diet with added β-glucan of three different molecular weights (1450, 730, and 370 kDa) did not differ among the groups [16]. Another report showed that all of the molecular weight forms of β-glucan (2348, 1311, 241, 56, 21 or <10 kDa) significantly reduced plasma cholesterol concentrations when compared with the control diet [17]. This discrepancy was explained by the experimental condition, such as excessive doses of β-glucan (6.8–8.4%). Our previous study showed that partially hydrolyzed barley β-glucan (50 and 110 kDa, 2.5% β-glucan in the diet) demonstrated the physiological functions similar to intact barley β-glucan which improved glucose and lipid metabolism [18].

Propionate, one of the major short-chain fatty acids (SCFAs), plays a significant role in the cholesterol-lowering effect [19]. A lot of evidence has accumulated to support the effect of β-glucan intake on cholesterol metabolism and gut microbiota metabolism, and it is clear that intake of β-glucans modifies the balance of gut microbiota [20]. Studies have shown that both cholesterol and bile acid metabolism is regulated by the metabolism of gut microbiota. A prebiotic effect was previously reported in humans: β-glucan altered the microbiota and led to an improvement in bile acid metabolism by the gut microbial community [21]. Generally, low molecular weight dietary fiber is more fermentable compared to high molecular weight fiber. However, an increase in SCFAs in the feces of subjects supplemented with high molecular weight barley β-glucan (HMW-BG) increased fecal bile acids [22]. The same effects were not observed in subjects supplemented with low molecular weight barley β-glucan (LMW-BG). In contrast to this result, a positive effect of β-glucans, such as promotion of fecal SCFAs, especially the low molecular weight form, was also reported in the colon tissue of both healthy and LPS-induced enteritis rats [23]. These results suggest that effects of low and high molecular weight β-glucans on their physiological functions were still controversial because of different experimental conditions.

We hypothesized that HMW-BG, with high viscosity, attenuates the glycemic response and lipid absorption, whereas LMW-BG, with high fermentability affects the glycemic response and lipid metabolism by prebiotic actions, such as SCFA production. The main objective of this study was to investigate the effect of LMW-BG on cecal fermentation, and glucose and lipid metabolism in mice fed a moderate-fat diet, compared with the effects of HMW-BG.

## 2. Materials and Methods

### 2.1. Chemical Analysis of β-Glucan

LMW-BG, partially hydrolyzed by cellulase, was obtained from ADEKA Corp. (30SP, Tokyo, Japan). HMW-BG was purchased from Megazyme Ltd. (Bray, Ireland). The average molecular weights of LMW-BG and HMW-BG were approximately 12 and 500 kDa, respectively. The total dietary fiber (TDF) content of LMW-BG and HMW-BG was determined to be 45% and 94%, respectively, using the method of Prosky et al. [24]. The β-glucan content of LMW-BG and HMW-BG was determined to be 33% and 94%, respectively, using the method of McCleary et al. (AOAC 995.16) [25].

### 2.2. Animals and Study Design

Male C57BL/6J 4-week-old mice were purchased from Charles River Laboratories Japan, Inc. (Yokohama, Japan). Each mouse was housed individually in polycarbonate cages. Mice were maintained on a 12 h light/dark cycle (lights on at 08:00 h). The studies were approved by the Otsuma Women’s University Animal Research Committee (Tokyo, Japan) and were performed in accordance with the Regulation on Animal Experimentation at Otsuma Women’s University (No.19007). After acclimatization for 7 days, the mice were randomized into 3 groups (*n* = 8 per group) and shifted to a 25% fat energy diet supplemented with LMW-BG or HMW-BG powder. LMW-BG and HMW-BG were added to the diets at concentrations corresponding to 4% β-glucan. The total dietary fiber content of the LMW-BG test diet was 5.48%; therefore, the other diets were supplemented with the amount of cellulose necessary to adjust the total dietary fiber content to 5.48%. The compositions of the experimental diets are shown in Table 1. Purified HMW-BG has poor solubility at body temperature; therefore, it was precipitated in ethanol and then dissolved in hot water, according to the manufacturer’s instruction. Dissolved HMW-BG (31.6%) was mixed with corn starch (68.4%), freeze-dried, and then the freeze-dried mixture was supplemented into the experimental diet. The protein and available carbohydrate contents in the LMW-BG diet were adjusted with casein and dextrinized corn starch, because the LMW-BG contained 4.2% protein and 33.6% available carbohydrate. Mice were fed the experimental diets ad libitum for 61 days. Food intake and body weights were monitored 3 times per week throughout the study period. Feces were collected for the final 5 days of the study period. The feces were freeze dried after washing the surface with distilled water to remove attached diet powder, milled, and kept at −20 °C until measurement. After fasting for 8 h, mice were sacrificed by isoflurane/CO_2_ anesthesia, then the cecum with digesta, adipose tissues (retroperitoneal, mesenteric, epididymal depot fats), and liver were dissected and weighed. Small samples of liver (200 mg) were suspended in RNA Stabilization Reagent (RNAlater, Qiagen, Hilden, Germany), and the remainder was freeze-dried, milled, and stored at −20 °C until required for cholesterol and triglyceride analysis. Small samples of ileum (40 mm portion from the cecum) were suspended in RNA Stabilization Reagent (RNAlater, Qiagen, Hilden, Germany). Cecum with digesta was stored at −20 °C until required for major microbiota and short chain fatty acid analysis. Blood samples, collected from the heart under isoflurane/CO_2_ anesthesia at sacrifice, were centrifuged, and serum was collected for biochemical analysis.

### 2.3. Biochemical Analysis of the Serum and Concentration of Liver Lipids

Total, LDL, and HDL cholesterol, triglycerides and non-esterified fatty acids (NEFA) were measured in mouse sera using Hitachi 71,870 auto-analyzers at the Nagahama Research Institute (Oriental Yeast Co., Ltd., Shiga, Japan). Serum leptin and insulin concentrations were measured using enzyme-linked immunosorbent assay (ELISA) kits (Mouse Leptin Immunoassay Kit, R&D Systems, Inc., MN, USA; Mouse Insulin ELISA Kit (TMB), Shibayagi Co., Ltd., Gunma, Japan). A 2:1 (*v*/*v*) chloroform–methanol solution was used to extract lipids from the liver [26], which were then dissolved in isopropanol containing 10% Triton X-100 (FUJIFILM Wako Pure Chemical Corporation, Osaka, Japan). Hepatic cholesterol and triglyceride levels were measured enzymatically with commercial kits (Cholesterol E-test and Triglyceride E-test; FUJIFILM Wako Pure Chemical Corporation, Osaka, Japan).

### 2.4. Total Fecal Lipid and β-Glucan Analysis

Fecal lipids were extracted using a 2:1 (*v*/*v*) chloroform–methanol mixture under acidic conditions (acetic acid was added to a final concentration of 4%) [26,27] and determined gravimetrically. Fecal β-glucans were analyzed to assess the fermentation rate according to the method of McCleary et al. (AOAC 995.16) [25].

### 2.5. Analysis of Short Chain Fatty Acids in Cecal Digesta

The concentration of cecal SCFAs was determined by the method previously described, using a gas chromatography–mass spectrometry (GC/MS) system [28]. GC/MS used a 7890B GC system (Agilent, Tokyo, Japan) equipped with a 5977A MSD (Agilent). A DB-5MS capillary column (30 m × 0.53 mm) (Agilent) was used to separate the SCFAs. SCFA concentrations were calculated by comparing their peak areas with the internal standard (crotonic acid) and were expressed as μmol/cecum.

### 2.6. Analysis of Major Microbiota in Cecal Digesta

Cecal bacterial counts were analyzed by real-time PCR according to the methods previously described [29,30]. DNA extraction from cecal digesta was performed by using a QIAamp Fast DNA Stool Mini Kit (QIAGEN GmbH). Two µl of DNA solution from 200 mg cecum digesta was used for the real-time PCR. Each real-time PCR analysis was performed in a 20 μL reaction mixture containing DNA and PowerUp SYBR Green Master Mix (Thermo Fisher Scientific, Waltham, MA, USA). Real-time PCR amplification and detection were performed in 96-well optical plates with a QuantStudio 3 real-time PCR system (Thermo Fisher Scientific K.K., MA, USA). A standard curve was generated with the real-time PCR data and the corresponding cell count, for dilution series of the following standard strains: *Bacteroides fragilis* JCM11019 (for *Bacteroides fragilis group*), *Prevotella melaninogenica* JCM6325 (for *Prevotella*), *Bifidobacterium longum* JCM1217 (for *Bifidobacterium*), *Lactobacillus rhamnosus* ATCC8530 (for *Lactobacillus*), *Clostridium coccoides* JCM1395T (for *Clostridium coccoides group*), *Ruminococcus albus* JCM14654 (for *Clostridium leptum subgroup*), *Collinsella aerofaciens* JCM10188 (for *Atopobium cluster*). Group-specific primers for the real-time PCR are shown in Supplementary Table S1. Colony formation units (CFU) were calculated using the standard curve for the representative strain of each group obtained as described above.

### 2.7. Expression Analysis of mRNAs in Liver and Ileum

Primer sequences are presented in Supplementary Table S2. Total RNAs in the liver and ileum were prepared using RNeasy mini kits (Qiagen, Hilden, Germany) according to the manufacturer’s instructions. mRNA expression was measured with a Quant3 Real-Time PCR System and PowerUp SYBR Green Master Mix (Thermo Fisher Scientific, Waltham, MA, USA) using cDNA prepared by RT-PCR. The reaction mixture for RT-PCR was prepared with 10 mM dNTPs (TOYOBO Co., Ltd., Osaka, Japan), 0.3 μg/mL Random primer (Life Technologies Japan, Tokyo, Japan), ReverTra ace Buffer (ReverTra ace, TOYOBO Co., Ltd., Osaka, Japan) and mixed into 5μg RNA/11.5μL RNAase-free water. The reaction was carried out by RT-PCR at 30 °C for 10 min, 42 °C for 60 min, and 99 °C for 5 min. The sample was diluted 20-fold with RNAase-free water to form cDNA template, which was used to measure mRNA expression. The 2^−ΔΔCT^ method was utilized for data analysis. The reference gene was 36B4. The ΔΔCT is the difference between the ΔCT for the BG diets and control diet. Relative expression levels are presented as fold changes to the control group (arbitrary unit).

### 2.8. Statistical Analysis

Sample sizes were determined from our previous study [18]. Twenty-four mice (8 mice per group) were used. Data are presented as mean ± standard error of the mean. Significant difference (*p* < 0.05) between group means was determined by Tukey–Kramer’s test or the Student’s *t*-test. The relationships among the SCFAs and parameters related to the prebiotic effect were assessed using Spearman’s rank correlation coefficient. JMP (Version 14.1, SAS Institute Inc., Cary, NC, USA) was used to perform the statistical analyses.

## 3. Results

### 3.1. Food Intake, Body Weight and Organ Weight

Body weight gain, food intake, and food efficiency ratio in mice fed LMW-BG or HMW-BG are shown in Table 2. Body weight gain was significantly lower in the HMW-BG group than the control group (*p* < 0.05); however, food intake was significantly lower in both LMW-BG and HMW-BG groups than the control group. A significant difference in the food efficiency ratio between the LMW-BG and HMW-BG group was observed (*p* < 0.05). The organ weights in mice fed LMW-BG and HMW-BG are shown in Table 3. The weights of the cecum with digesta were significantly higher in both LMW-BG and HMW-BG groups compared with the control group (*p* < 0.05). Liver and total abdominal, retroperitoneal, epididymal and mesenteric fat weights were significantly lower in the HMW-BG group compared with the control group (*p* < 0.05).

### 3.2. Fecal Total Fat Excretion and Apparent Absorption of Fat

Fecal total fat excretion and apparent digestibility of fat in the final five days are shown in Table 4. The apparent digestibility of fat in the HMW-BG group was significantly lower than the control group. The same tendency was observed in the LMW-BG group, but the difference was not significant.

### 3.3. Fecal β-Glucan Excretion and Fermentability of β-Glucan

**Figure 1:**
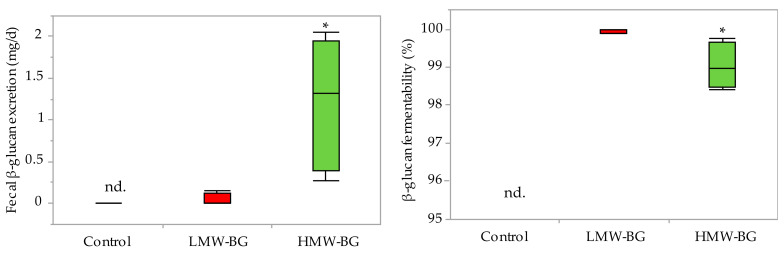
Box-and-whisker plots of fecal β-glucan excretion and the fermentability of β-glucan. Box marked by an asterisk differs significantly compared to the LMW-BG group (Student’s *t*-test, *p < 0.05). nd., not detected; LMW-BG, low molecular weight β-glucan; HMW-BG, high molecular weight β-glucan.

Fecal β-glucan excretion and fermentability of β-glucan are shown in Figure 1. Fecal β-glucan was significantly higher in the HMW-BG group than the LMW-BG group. Fecal β-glucan was not detected in the control group. The fermentability of β-glucan in the LMW-BG group was almost 100%, and a significant difference was observed when compared with the HMW-BG group.

### 3.4. Short-Chain Fatty Acid (SCFA) Concentrations in Cecal Digesta

**Figure 2:**
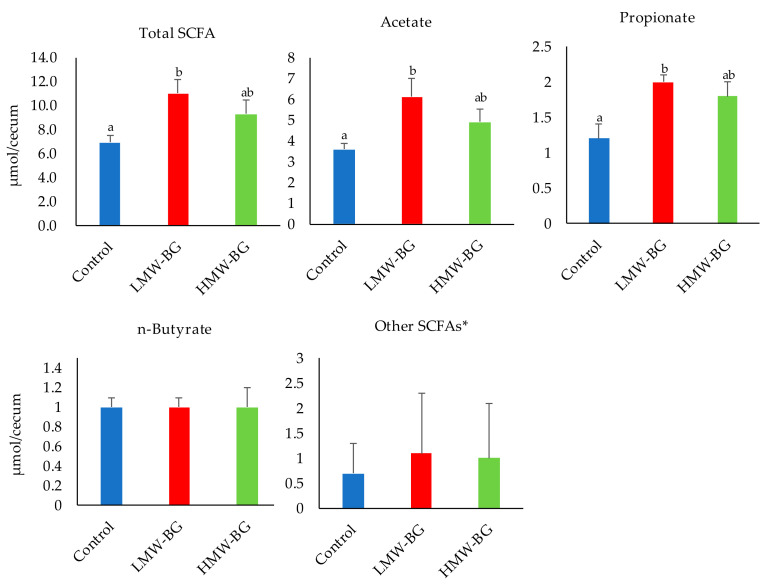
Short-chain fatty acid (SCFA) concentrations in the cecal digesta of mice fed the test diets. Bars represent means and SE, *n* = 8. * Other SCFAs, the sum of the concentrations of formate, iso-butyrate, iso-valerate, and valerate is shown. Means with suffixed superscript letters differ significantly (Tukey–Kramer’s test, *p* < 0.05). LMW-BG; low molecular weight β-glucan, HMW-BG; high molecular weight β-glucan.

The concentrations of short-chain fatty acids in cecal digesta are shown in Figure 2. Total SCFA concentrations, as well as acetate and propionate concentrations, were significantly higher in the LMW-BG group than the control group. The same tendency was observed in the HMW-BG group, but the difference was not significant.

### 3.5. Bacterial Counts of the Major Microbiota Groups in the Cecal Digesta of Mice Fed the Test Diets

**Figure 3:**
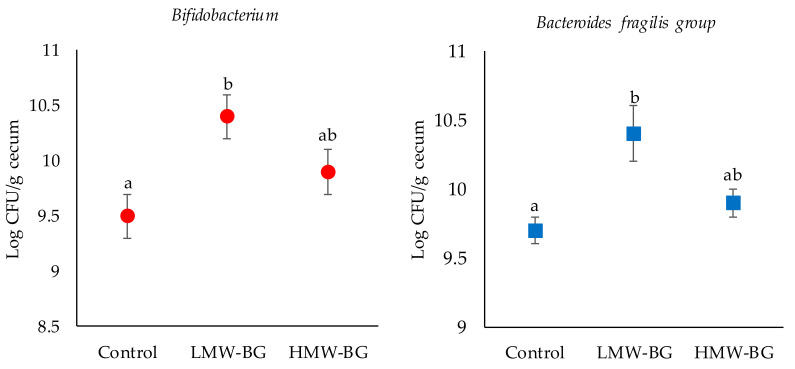
Bacterial counts of Bifidobacterium and Bacteroides fragilis group in the cecal digesta of mice fed the test diets. Dots and error bars represent means and SE, *n* = 8. Means with suffixed superscript letters differ significantly (Tukey–Kramer’s test, *p* < 0.05). LMW-BG; low molecular weight β-glucan, HMW-BG; high molecular weight β-glucan.

Bacterial counts of the major microbiota groups in the cecal digesta are shown in Figure 3 and Supplementary Table S3. Bacterial counts of *Bifidobacterium* were significantly higher in the LMW-BG group than the control group. Bacterial counts of *Bacteroides fragilis group* were significantly higher in the LMW-BG group than the control and HMW-BG groups. No significant differences in the other bacterial counts were observed.

### 3.6. Biochemical Analysis of the Serum and Liver Lipids

Serum biochemical concentrations are shown in Table 5. Serum total- and LDL-cholesterol and leptin concentrations were significantly reduced in both BG groups compared with the control (*p* < 0.05). Serum HDL-cholesterol concentration was also significantly lower in the LMW-BG group compared with the control group (*p* < 0.05). Serum glucose concentration was significantly lower in the LMW-BG group than the control group, whereas serum insulin concentration was significantly lower in the HMW-BG group than the control group (*p* < 0.05). Serum NEFA concentration was significantly lower in the HMW-BG group than the LMW-BG group (*p* < 0.05), whereas no significant difference was observed between the control and BG groups. There was no significant difference in serum triglyceride concentration among the groups. Liver lipid levels are shown in Supplementary Table S4: cholesterol and triglyceride accumulation (mmol/liver) and triglyceride concentration (mmol/g liver) were not statistically different among the groups.

### 3.7. Expression of mRNAs Related to Liver Lipid Metabolism

**Figure 4:**
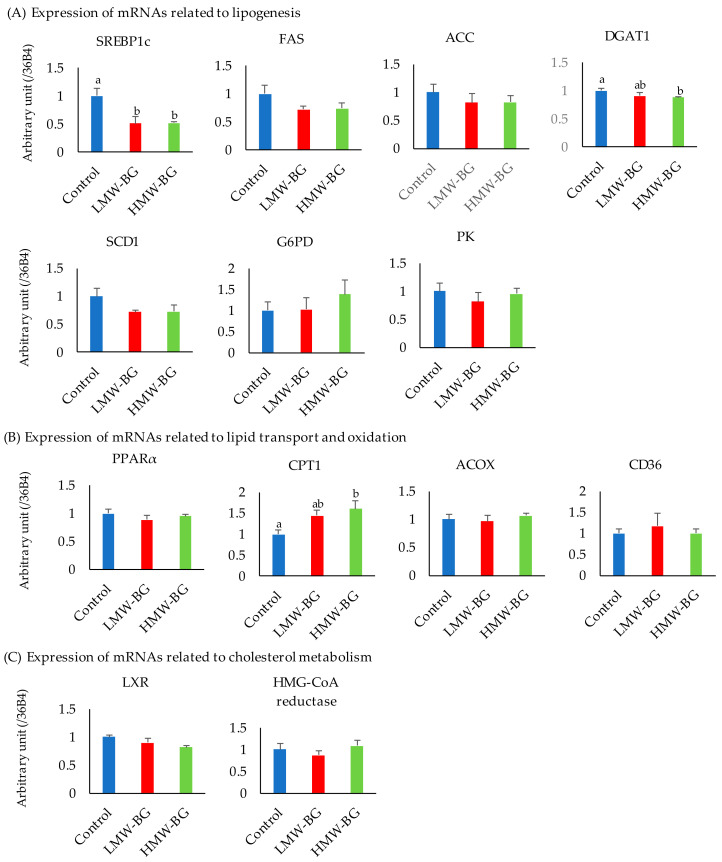
The expression of mRNAs related to lipogenesis (**A**), lipid transport and oxidation (**B**), and cholesterol metabolism (**C**) in liver. Bars represent means and SE, *n* = 8. Means with suffixed superscript letters differ significantly (Tukey–Kramer’s test, *p* < 0.05). LMW-BG; low molecular weight β-glucan, HMW-BG; high molecular weight β-glucan. SREBP-1c, sterol regulatory element-binding protein-1c; FAS, fatty acid synthase; ACC, acetyl-CoA carboxylase; DGAT1, diacyl glycerol acyl-transferase 1; SCD1, stearoyl coenzyme A desaturase 1; G6PD, glucose 6-phosphate dehydrogenase; PK, pyruvate kinase; PPPARα, peroxisome proliferator-activated receptorα; CPT1, carnitine palmitoyl transferase 1; ACOX, acyl-coenzyme A oxidase; CD36 (FAT), fatty acid translocase; LXR, liver X receptor; 3-hydroxy-3-methyl-glutaryl-CoA reductase, (HMG-CoA reductase).

Hepatic mRNA expression levels are shown in Figure 4. The mRNA expression level of sterol regulatory element-binding protein-1c (SREBP-1c) was significantly lower in both BG groups when compared with the control group (*p* < 0.05). No significant differences in the mRNA expression levels of peroxisome proliferator-activated receptorα (PPARα) and liver X receptor (LXR) were observed. The mRNA expression level of diacyl glycerol acyl-transferase 1 (DGAT1) was significantly lower in the HMW-BG group than the control group, whereas the mRNA expression level of carnitine palmitoyl transferase 1 (CPT1) was significantly higher in the HMW-BG group when compared with the control group (*p* < 0.05). No significant differences were observed in the mRNA expression levels of glucose 6-phosphate dehydrogenase (G6PD), pyruvate kinase (PK), fatty acid synthase (FAS), acetyl-CoA carboxylase (ACC), acyl-coenzyme A oxidase (ACOX), stearoyl coenzyme A desaturase 1 (SCD1), fatty acid translocase (CD36), and 3-hydroxy-3-methyl-glutaryl-CoA reductase, (HMG-CoA reductase).

### 3.8. Expression of mRNAs Related to Ileal L Cell Function

**Figure 5:**
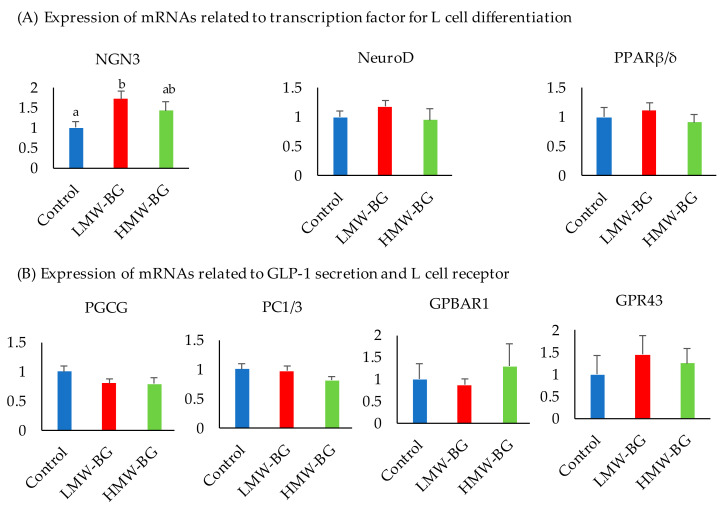
Expression of mRNAs related to transcription factor for L cell differentiation (**A**) and GLP-1 secretion and L cell receptor (**B**) in ileum. Bars represent means and SE, *n* = 8. Means with suffixed superscript letters differ significantly (Tukey–Kramer’s test, *p* < 0.05). LMW-BG; low molecular weight β-glucan, HMW-BG; high molecular weight β-glucan. NGN3, neurogenin 3; Neuro D, neurogenic differentiation factor; PPPARβ/δ, peroxisome proliferator-activated receptorβ/δ; PGCG, proglucagon; PC1/3, prohormone convertases1/3; GPBAR1, G-protein-coupled bile acid receptor 1; GPR43, G-protein-coupled receptor 43.

Ileal mRNA expression levels related to L cell function are shown in Figure 5. The mRNA expression level of neurogenic differentiation factor (NGN3) was significantly higher in the LMW-BG group when compared with the control group (*p* < 0.05). No significant differences were observed in the mRNA expression levels of neurogenic differentiation factor (Neuro D), prohormone convertases1/3 (PC1/3), proglucagon (PGCG), G-protein-coupled bile acid receptor 1 (GPBAR1), and G-protein-coupled receptor 43 (GPR43).

## 4. Discussion

The effects of LMW-BG and HMW-BG on cecal fermentation, glucose and lipid metabolism in mice fed a moderate-fat diet were compared. HMW-BG specific effects were observed in inhibiting dietary fat absorption and reducing abdominal depot fat. LMW-BG specific effects were observed in increasing bacterial counts of *Bifidobacterium* and *Bacteroides*, and consequently increasing cecal total SCFAs, acetate, and propionate. mRNA expression of NGN3, an L cell marker, was increased in the LMW-BG group compared with the control group. These results indicate that LMW-BG has the potential to improve glucose and lipid metabolism by a different mechanism from HMW-BG. Many studies have reported that HMW-BG has a greater impact than LMW-BG on the improvement of glucose and lipid metabolism; however, our results suggested that LMW-BG improves glucose and lipid metabolism through prebiotic effects. The prebiotic effects are expected to protect from pro-inflammation of organs and promote the gut immune system. Further studies are needed to clarify the prebiotic effects of LMW-BG.

Similar significant reductions in the mRNA expression of SREBP-1c and serum total- and LDL-cholesterol and leptin concentrations were observed in both BG groups compared with the control group. LMW-BG was almost 100% fermented and cecal bacterial counts of *Bifidobacterium* and *Bacteroides* were significantly increased in the LMW-BG group, resulting in an increase in the cecal contents of acetic acid and propionic acid. The results indicated that decreases in abdominal depot fats, serum cholesterol and leptin concentrations in the HMW-BG group were caused by the inhibition of nutrient absorption due to high viscosity in the digestive tract, whereas the changes in the LMW-BG group were due to prebiotic effects. It is reported that an increase in SCFAs decreases serum cholesterol, fasting blood glucose, and leptin concentrations [19,21]. The blood was collected under fasting conditions in this study; therefore, the serum GLP-1 concentration was not measured. NGN3 is a key factor which initiates endocrine differentiation [31]. NGN3 and BETA2/Neuro D (Neuro D) specifically induce certain types of enteroendocrine cells, such as L cells [32]. It is therefore possible that the serum glucose concentration in the LMW-BG group might be lowered through the action of GLP-1. Spearman’s rank correlation coefficient analysis related to the prebiotic effect is shown in Supplementary Table S5. Significant positive correlation coefficients between cecal total SCFAs, especially acetate and propionate, and serum biomarkers were observed. It is suggested that serum glucose and lipid concentrations were improved by the SCFAs, directly or indirectly. Significant positive correlation coefficients between cecal SCFAs and the mRNA expression of ileal NGN3 were also observed. Furthermore, significant positive correlation coefficients between ileal mRNA expressions of NGN3, NeuroD, and GPBAR1 were observed (Supplementary Table S5); the increases in mRNA levels might decrease serum glucose and lipid concentrations through an L cell function. It was reported that GPBAR1 is a selective regulator of intestinal L cell differentiation [33]. Further studies are needed to elucidate the mechanism of LMW-BG on glucose metabolism through L cell function. HMW-BG had a high fermentation rate but the microbiota influence and the amount of SCFAs were relatively small when compared with LMW-BG, suggesting that the high viscosity of HMW-BG contributes more than the prebiotic effect.

Significant reductions in food intake were observed in both BG groups. HMW-BG increases viscosity, thereby delaying gastric emptying time [34], suggesting that the reduction in food intake in the HMW-BG group in this study might be due to delayed gastric emptying. The reduction in food intake in the LMW-BG group might be caused by a different mechanism, such as intestinal hormone secretion which is promoted by SCFAs. It was reported that propionate in the colon of rats and mice stimulated the release of both glucagon-like peptide 1 (GLP-1) and peptide PYY, which are anorexigenic gut hormones [35]. Anorexigenic gut hormones related to food intake were not analyzed in our study. Further studies are needed to elucidate the mechanism of reduction in food intake in the LMW-BG and HMW-BG groups.

Serum insulin concentrations were significantly reduced in the HMW-BG group only; however, hepatic mRNA expression of SREBP-1c controlled by insulin was significantly reduced in both groups. Hepatic SREBP-1c has been reported as an insulin-mediated transcriptional activator of genes involved in carbohydrate and lipid metabolism [36,37]. The reduction in SREBP-1c mRNA expression in the HMW-BG group was caused by decreasing insulin secretion through the suppression of the digestion and absorption of carbohydrates. Fasting serum glucose concentrations were decreased in the LMW-BG group; therefore, the mRNA expression of SREBP-1c was decreased through the modification of glucose metabolism by the secretion of incretins such as GLP-1, whose secretion was enhanced by SCFAs. In this study, there were no significant differences in mRNA expression levels of lipogenic enzymes, such as FAS and ACC, among the groups; however, we suggest that there was an effect on lipid metabolism through suppression of SREBP-1c mRNA expression. In our previous study using diet-induced obesity mice, we confirmed a decrease in the mRNA expression of fatty acid synthase and glucose-6-phosphate dehydrogenase due to a reduction in SREBP-1c mRNA expression [38]. Significant negative correlation between cecal SCFAs and mRNA expression of hepatic SREBP-1c, and significant positive correlation between mRNA expression of hepatic SREBP-1c and FAS, CD36, LXR, PPARα was observed (Supplementary Table S6). It is concluded that the regulation of lipid metabolism mediated by SREBP-1c is exerted in HMW-BG and LMW-BG by different mechanisms.

A previous study using barley flour with high β-glucan reported different results [39]: the abundance of *Bacteroides* was significantly higher in the β-glucan rich barley flour group compared with the β-glucan free barley flour group, whereas the abundance of *Clostridium* clusters was significantly lower in the β-glucan rich barley flour group compared with the β-glucan free barley flour group. We suggest that the food matrix is responsible for the differences between β-glucan rich barley flour and isolated β-glucan because it affects the release of carbohydrates and β-glucan, thereby affecting the digestibility and absorption rate. Consumption of barley flour leads to the slow release of carbohydrates and β-glucan, which would promote reductions in a postprandial glucose rise and serum cholesterol concentration. Barley flour also contains arabinoxylan which has a high prebiotic effect [40]. It is reported that arabinoxylan from wheat modulates both the gut microbiota and lipid metabolism in high-fat diet-induced obese mice [41]. When barley flour is ingested, it is expected that the combination of β-glucan and arabinoxylan in barley would have a synergistic affect.

## 5. Conclusions

Our results indicated that LMW-BG and HMW-BG affect glucose and lipid metabolism by different mechanisms. The results from this study and previous reports also suggested that the physiological responses to ingested β-glucan rich barley flour or isolated HMW-BG differed. The prebiotic effect of LMW-BG is expected to be applied to several foods and beverages. It was revealed that the expected function of barley β-glucan differs according to the molecular weight; therefore, it is suggested that different nutritional interventions may be possible, depending on the purpose of the treatment.

## Figures and Tables

**Table 1:** Composition of the experimental diets.

			(g/kg Diet)
	Control	LMW-BG	HMW-BG
Casein	200	183	200
L-cystin	3	3	3
Corn starch	350.7	300.9	350.7
Dextrinized corn starch	132	132	45.4
Sucrose	100	100	100
Soybean oil	70	70	70
Lard	42	42	42
Cellulose	54.8	-	14.8
LMW-BG	-	121.6	-
HMW-BG mixed with corn starch	-	-	126.6
AIN-93G mineral mixture	35	35	35
AIN-93 vitamin mixture	10	10	10
Choline bitartrate	2.5	2.5	2.5
*t*-Butylhydroquinone	0.014	0.014	0.014

LMW-BG: Low molecular weight β-glucan; 32.9%, total dietary fiber (TDF); 45.1%. HMW-BG mixed with corn starch was prepared from a freeze-dried mixture of previously resolved high molecular weight β-glucan (31.6%) in hot water and corn starch (68.4%).

**Table 2:** Body weight gain, food intake, and food efficiency ratio.

	Control	LMW-BG	HMW-BG
Initial weight (g)	20.6 ± 0.5	20.6 ± 0.4	20.6 ± 0.4
Final weight (g)	31.3 ± 0.9	31.0 ± 0.8	28.9 ± 0.7
Body weight gain (g/d)	0.19 ± 0.01 ^a^	0.17 ± 0.01 ^ab^	0.14 ± 0.01 ^b^
Food intake (g/d)	4.1 ± 0.2 ^a^	3.2 ± 0.1 ^b^	3.4 ± 0.2 ^ab^
Food efficiency ratio (%)	4.56 ± 0.23 ^ab^	5.30 ± 0.25 ^a^	4.01 ± 0.22 ^b^

Values are means ± SE, *n* = 8. Means with suffixed superscript letters differ significantly (Tukey–Kramer’s test, *p* < 0.05). LMW-BG; low molecular weight β-glucan, HMW-BG; high molecular weight β-glucan.

**Table 3:** Organ weights.

	Control	LMW-BG	HMW-BG
Liver (g)	1.11 ± 0.03 ^a^	1.04 ± 0.05 ^ab^	0.92 ± 0.02 ^b^
Cecum with digesta (g)	0.30 ± 0.03 ^a^	0.51 ± 0.03 ^b^	0.44 ± 0.03 ^b^
Total abdominal fat	2.09 ± 0.11 ^a^	1.54 ± 0.23 ^ab^	1.25 ± 0.19 ^b^
Retroperitoneal fat (g)	0.46 ± 0.03 ^a^	0.32 ± 0.06 ^ab^	0.25 ± 0.05 ^b^
Epididymal fat (g)	1.22 ± 0.06 ^a^	0.89 ± 0.08 ^ab^	0.75 ± 0.10 ^b^
Mesenteric fat (g)	0.42 ± 0.03 ^a^	0.33 ± 0.05 ^ab^	0.26 ± 0.05 ^b^

Values are means ± SE, *n* = 8. Means with suffixed superscript letters differ significantly (Tukey–Kramer’s test, *p* < 0.05). LMW-BG; low molecular weight β-glucan, HMW-BG; high molecular weight β-glucan.

**Table 4:** Fecal fat excretion and apparent digestibility of fat in the final 5 days.

	Control	LMW-BG	HMW-BG
Fat intake (mg/d)	404 ± 1 ^a^	339 ± 1 ^b^	344 ± 1 ^b^
Fecal fat excretion (mg/d)	11 ± 1 ^a^	21 ± 3 ^ab^	39 ± 10 ^b^
Apparent digestibility of fat (%)	97.2 ± 0.2 ^a^	93.8 ± 0.7 ^ab^	88.2 ± 3.2 ^b^

Values are means ± SE, *n* = 8. Means with suffixed superscript letters differ significantly (Tukey–Kramer’s test, *p* < 0.05). LMW-BG; low molecular weight β-glucan, HMW-BG; high molecular weight β-glucan.

**Table 5:** Serum biochemical concentrations.

	Control	LMW-BG	HMW-BG
Total cholesterol (mmol/L)	3.42 ± 0.09 ^a^	2.57 ± 0.23 ^b^	2.66 ± 0.12 ^b^
LDL-cholesterol (mmol/L)	0.15 ± 0.01 ^a^	0.09 ± 0.01 ^b^	0.09 ± 0.01 ^b^
HDL-cholesterol (mmol/L)	1.88 ± 0.05 ^a^	1.52 ± 0.15 ^b^	1.54 ± 0.07 ^ab^
Triglyceride (mmol/L)	0.52 ± 0.05	0.45 ± 0.07	0.31 ± 0.04
NEFA (μmol/L)	635.9 ± 23.1 ^ab^	654.9 ± 36.6 ^a^	533.5 ± 37.1 ^b^
Glucose (mmol/L)	21.1 ± 0.69 ^a^	17.21 ± 1.17 ^b^	18.64 ± 0.78 ^ab^
Insulin (ng/mL)	1.20 ± 0.17 ^a^	1.05 ± 0.24 ^ab^	0.39 ± 0.13 ^b^
Leptin (ng/mL)	15.30 ± 1.29 ^a^	9.58 ± 2.05 ^b^	5.74 ± 1.19 ^b^

Values are means ± SE, *n* = 8. Means with suffixed superscript letters differ significantly (Tukey–Kramer’s test, *p* < 0.05). LMW-BG; low molecular weight β-glucan, HMW-BG; high molecular weight β-glucan, NEFA; non-esterified fatty acid.

## Data Availability

Data are contained within the article or Supplementary Materials.

## Supplementary Materials

The following are available online at https://www.mdpi.com/2072-6643/13/1/130/s1. Table S1: Group-specific primers for real-time reverse transcription polymerase chain reaction (PCR), Table S2: Primers used in the real-time reverse transcription polymerase chain reaction (PCR), Table S3: Bacterial counts of major genus microbiota in the cecal digesta of mice fed the test diets, Table S4: Liver lipid accumulation, Table S5: Spearman’s rank correlation coefficient related to the prebiotic effect, Table S6: Spearman’s rank correlation coefficients for the relationship between parameters related to liver lipid metabolism (A) and ileal L cell function (B).

## Abbreviations

LMW-BG, low molecular weight β-glucan; HMW-BG, high molecular weight β-glucan; NEFA, non-esterified fatty acid; SCFA, short-chain fatty acid; SREBP-1c, sterol regulatory element-binding protein-1c; FAS, fatty acid synthase; ACC, acetyl-CoA carboxylase; DGAT1, diacyl glycerol acyl-transferase 1; SCD1, stearoyl coenzyme A desaturase 1; G6PD, glucose 6-phosphate dehydrogenase; PK, pyruvate kinase; PPPARα, peroxisome proliferator-activated receptorα; CPT1, carnitine palmitoyl transferase 1; ACOX, acyl-coenzyme A oxidase; CD36 (FAT), fatty acid translocase; LXR, liver X receptor; 3-hydroxy-3-methyl-glutaryl-CoA reductase, (HMG-CoA reductase); NGN3, neurogenin 3; Neuro D, neurogenic differentiation factor; PPPARβ/δ, peroxisome proliferator-activated receptorβ/δ; PGCG, proglucagon; PC1/3, prohormone convertases1/3; GPBAR1, G-protein-coupled bile acid receptor 1; GPR43, G-protein-coupled receptor 43.
