# Supplementary material for: Low Molecular Weight Barley β-Glucan Affects Glucose and Lipid Metabolism by Prebiotic Effects
Source: Nutrients. 2020 Dec 31;13(1):130. doi: 10.3390/nu13010130 (PMC7823751; doi:10.3390/nu13010130)
Supplement: Supplementary file 1 [file nutrients-13-00130-s001.pdf]

Supplementary Table S1. Group-specific primers for real time PCR

		primer sequence	Standard bacteria	Strain No.
Bacteroides fragilis group	Forward	GTCAGTTGTGAAAGTTTGC	*Bacteroides fragilis*	JCM11019
	Reverse	CAATCGGAGTTCTTCGTG		
Prevotella	Forward	CACCAAGGCGACGATCA	*Prevotella melaninogenica*	JCM6325
	Reverse	GGATAACGCCYGGACCT		
Bifidobacterium	Forward	AGGGTTCGATTCTGGCTCAG	*Bifidobacterium longum*	JCM1217
	Reverse	CATCCGGCATTACCACCC		
Lactobacillus	Forward	TGGAAACAGGTGCTAATACCG	*Lactobacillus rhamnosus*	ATCC8530
	Reverse	GTCCATTGTGGAAGATTCCC		
Clostridium coccoides group	Forward	AAATGACGGTACCTGACTAA	*Clostridium coccoides*	JCM1395T
	Reverse	CTTTGAGTTTCATTCTTGCGAA		
Clostridium leptum subgroup	Forward	GCACAAGCAGTGGAGT	*Ruminococcus albus*	JCM14654
	Reverse	CTTCCTCCGTTTTGTCAA		
Atopobium cluster	Forward	GGGTTGAGAGACCGACC	*Collinsella aerofaciens*	JCM10188
	Reverse	CGGRGCTTCTTCTGCAGG		

Supplementary Table S2. Primers used in the real-time reverse transcription polymerase chain reaction (PCR).

	Forward	Reverse
Hepatic Lipid metabolism		
PPARα	5'-AGGAAGCCGTTCTGTGACAT-3'	5'-AATCCCCTCCTGCAACTTCT-3'
LXR	5'-CCTTCCTCAAGGACTTCAGTTACAA-3'	5'-CATGGCTCTGGAGAACTCAAAGAT-3'
SREBP1c	5'-GGCACTAAGTGCCCTCAACCT-3'	5'-GCCACATAGATCTCTGCCAGTGT-3'
FAS	5'-CCTGGATAGCATTCCGAACCT-3'	5'-AGCACATCTCGAAGGCTACACA-3'
ACC	5'-GGATGACAGGCTTGCAGCTATG-3'	5'-GGAACGTAAGTCGCCGGATG-3'
G6PD	5'-CTGGAACCGCATCATCGTGGAG-3'	5'-CCTGATGATCCCAAATTCATCAAAATAG-3'
SCD1	5'-CCTTATCATTGCCAACACCAT-3'	5'-AGCCAACCCACGTGAGAGAA-3'
CD36 (FAT)	5'-TCCCTCTCTGGAGTTCTTGG-3'	5'-TTGCAGCTGAGCAGAAAGAG-3'
PK	5'-TGCAATTGGAGCCGTGGA-3'	5'-TGGCTGTCATTCAAG-3'
DGAT1	5'-GCTGAGTCTGTCACCTACTTTTGG-3'	5'-AGGCTTGTAGAAGTGTCTGATGCA-3'
CPT1	5'-GATGGAGAGGATGTTCAACACTACAC-3'	5'-AGCCCTCATAGAGCCAGACCTT-3'
ACOX	5'-CAGCGTTACGAGGTGGCTGTTA-3'	5'-TGCCCAAGTGAAGGTCCAAAG-3'
HMG-CoA reductase	5'-CCAAGGTGGTGAGAGAGGTGTT-3'	5'-CGTCAACCATAGCTTCCGTAGTT-3'
Ileal L cell function		
NeuroD	5'-CTTGGCCAAGAACTACATCTGG-3'	5'-CGTGTTTGAAAGAGAAGTTGCC-3'
NGN3	5'-AAGAGCGAGTTGGCACTCAGC-3'	5'-AAGCTGTGGTCCGCTATGCG -3'
PPARβ/δ	5'-ACTTGGCGTGGCGCCTGC-3'	5'-AGCGGTGTGGGTATGCGCA-3'
PGCG	5'-ATTGCCAAACGTCATGATGA-3'	5'-GGCGACTTCTTCTGGGAAGT-3'
PC1/3	5'-AGACAGCATTACACCATCTCTA-3'	5'-AGAACACTTCTCTGCATACCAAGGT-3'
GPBAR1	5'-AACGCTACATGGCAGTGTTG-3'	5'-GGAGGCCATAAACTTCCAGGTAGA-3'
GPR43	5'-GGGATCTGGGTCACATGCTTAT-3'	5'-ATGTCAGACAGACGGGTACCAA-3'
Reference		
36B4	5'-GGCCCTGCACTCTCGCTTTC-3'	5'-TGCCAGGACGCGCTTGT-3'

R =A or G

PPARα, peroxisome proliferator-activated receptorα; LXR, liver X receptor; SREBP-1c, sterol regulatory element-binding transcription factor-1c; FAS, fatty acid synthase; ACC, acetyl-CoA carboxylase; G6PD, glucose 6-phosphate dehydrogenase; SCD1, stearoyl coenzyme A desaturase 1; CD36 (FAT), fatty acid translocase; PK, pyruvate kinase; DGAT1, diacyl glycerol acyl-transferase 1; CPT1, carnitine palmitoyl transferase 1; ACOX, acyl-coenzyme A oxidase; 3-hydroxy-3-methyl-glutaryl-CoA reductase, (HMG-CoA reductase); NeuroD, neurogenic differentiation factor; NGN3, neurogenin 3; PPARβ/δ, peroxisome proliferator-activated receptorβ/δ; PGCG, proglucagon; PC1/3, prohormone convertases1/3; GPBAR1, G-protein–coupled bile acid receptor 1; GPR43, G-protein-coupled receptor 43; 36B4, acidic ribosomal protein.

Supplementary Table S3. Bacterial counts of major genus microbiota in the cecal digesta of mice fed the test diets

Log CFU/g cecum	Control	LMW-BG	HMW-BG
Lactobacillus	9.3±0.2	10.0±0.2	9.7±0.2
Prevotella	6.5±0.2	6.2±0.1	6.1±0.1
Clostridium coccoides group	9.1±0.1	9.2±0.1	9.2±0.1
Clostridium leptum subgroup	10.8±0.1	11.0±0.1	11.0±0.1
Atopobium cluster	9.1±0.2	9.7±0.2	9.3±0.2

Values are means±SE, n=8.

Supplementary Table S4. Liver lipid accumulation

	Control	LMW-BG	HMW-BG
Cholesterol (mmol/liver)	0.37±0.02	0.35±0.01	0.33±0.01
(mmol/g liver)	0.34±0.01	0.34±0.01	0.36±0.02
Triglyceride (mmol/liver)	2.18±0.21	2.44±0.18	2.08±0.27
(mmol/g liver)	1.98±0.16	2.38±0.19	2.22±0.24

Values are means±SE, n=8.

Supplementary Table S5. Spearman's rank correlation coefficient related to the prebiotic effect

	Ileum		Liver					Serum			
	NGN3	GPBAR1	SREBP-1c	FAS	CD36	LXR	PPARα	Total cholesterol	LDL-cholesterol	Glucose	Leptin
Total SCFAs	0.47*	0.22	-0.50*	0.08	-0.31	-0.45*	-0.31	-0.46*	-0.44*	-0.48*	-0.46*
Acetate	0.45*	0.20	-0.51*	0.15	-0.25	-0.44*	-0.31	-0.49*	-0.51*	-0.49*	-0.44*
Propionate	0.56*	0.37	-0.40	0.09	-0.22	-0.22	-010	-0.50*	-0.46*	-0.40	-0.41

* p<0.05

SCFA, short chain fatty acids; NGN 3, neurogenin 3; SREBP-1c, sterol regulatory element-binding transcription factor 1c; GPBAR1, G-protein–coupled bile acid receptor 1; FAS, fatty acid synthase; CD36 (FAT), fatty acid translocase; LXR, liver X receptor; PPARα, peroxisome proliferator-activated receptorα

Supplementary Table S6. Spearman's rank correlation coefficients for the relationship between parameters related to liver lipid metabolism (A) and ileal L cell function (B).

(A)	PPARα	LXR	FAS	ACC	G6PD	SCD1	CD36	PK	DGAT1	CPT1	ACOX	HMG-CoA reductase
SREBP-1c	0.42*	0.50*	0.46*	0.15	-0.02	0.17	0.42*	0.05	0.30	-0.16	0.39	0.30
PPARα		0.54*	0.39	0.56*	-0.16	0.34	0.34	0.57*	0.45*	0.06	0.88*	0.33
LXR			0.27	0.32	0.09	0.35	0.32	0.44*	0.76*	-0.45*	0.46*	-0.00
FAS				0.20	-0.03	0.41*	-0.01	0.31	0.05	-0.10	0.35	0.56*
ACC					-0.04	0.33	0.22	0.51*	0.35	0.15	0.45*	0.32
G6PD						-0.15	0.17	0.29	-0.04	-0.26	-0.04	0.18
SCD1							0.14	0.17	0.14	-0.50*	0.28	0.22
CD36								0.24	0.26	0.23	0.31	0.04
PK									0.43*	-0.03	0.55*	0.36
DGAT1										-0.34	0.39	-0.21
CPT1											0.08	0.17
ACOX												0.50*

(B)	NGN3	NeuroD	PPARβ/δ	PGCG	PC1/3	GPBAR1
NeuroD	0.50*					
PPARβ/δ	0.31	0.55*				
PGCG	0.13	0.52*	0.25			
PC1/3	0.26	0.44*	0.56*	0.38		
GPBAR1	0.64*	0.45*	0.29	0.24	0.26	
GPR43	0.39	0.34	0.45*	0.24	0.29	0.55*

SREBP-1c, sterol regulatory element-binding transcription factor-1c; PPARα, peroxisome proliferator-activated receptorα; LXR, liver X receptor; FAS, fatty acid synthase; ACC, acetyl-CoA carboxylase; G6PD, glucose 6-phosphate dehydrogenase; SCD1, stearoyl coenzyme A desaturase 1; CD36 (FAT), fatty acid translocase; PK, pyruvate kinase; DGAT1, diacyl glycerol acyl-transferase 1; CPT1, carnitine palmitoyl transferase 1; ACOX, acyl-coenzyme A oxidase; 3-hydroxy-3-methyl-glutaryl-CoA reductase, (HMG-CoA reductase); NeuroD, neurogenic differentiation factor; NGN3, neurogenin 3; PPARβ/δ, peroxisome proliferator-activated receptorβ/δ; PGCG, proglucagon; PC1/3, prohormone convertases1/3; GPBAR1, G-protein–coupled bile acid receptor 1; GPR43, G-protein-coupled receptor 43.
